# Novel nomogram to predict risk of bone metastasis in newly diagnosed thyroid *carcinoma*: a population-based study

**DOI:** 10.1186/s12885-020-07554-1

**Published:** 2020-11-03

**Authors:** Yuexin Tong, Chuan Hu, Zhangheng Huang, Zhiyi Fan, Lujian Zhu, Youxin Song

**Affiliations:** 1 Department of Orthopedic, Affiliated Hospital of Chengde Medical University, No. 36 Nanyingzi St, Chengde, 067000 Hebei China; 2 Qingdao University Medical College, Qingdao, 266000 Shandong China; 3 The First Affiliated Hospital of Wenzhou Medical University, Wenzhou, 325000 Zhejiang China

**Keywords:** T*hyroid carcinoma*, bone, metastasis, risk factors, Nomogram, SEER database

## Abstract

**Background:**

The aim of this study was to develop and validate a visual nomogram for predicting the risk of bone metastasis (BM) in newly diagnosed *thyroid carcinoma (*TC) patients.

**Methods:**

The demographics and clinicopathologic variables of TC patients from 2010 to 2015 in the Surveillance, Epidemiology and End Results (SEER) database were retrospectively reviewed. *Chi*-*squared* (χ2) test and logistic regression analysis were performed to identify independent risk factors. Based on that, a predictive nomogram was developed and validated for predicting the risk of BM in TC patients. The C-index was used to compute the predictive performance of the nomogram. Calibration curves and decision curve analysis (DCA) were furthermore used to evaluate the clinical value of the nomogram.

**Results:**

According to the inclusion and exclusion criteria, the data of 14,772 patients were used to analyze in our study. After statistical analysis, TC patients with older age, higher T stage, higher N stage, poorly differentiated, follicular thyroid carcinoma (FTC) and black people had a higher risk of BM. We further developed a nomogram with a C-index of 0.925 (95%CI,0.895–0.948) in the training set and 0.842 (95%CI,0.777–0.907) in the validation set. The calibration curves and decision curve analysis (DCA) also *demonstrate*d the reliability and accuracy of the clinical prediction model.

**Conclusions:**

The present study developed a visual nomogram to accurately identify TC patients with high risk of BM, which might help to further provide more individualized clinical decision guidelines.

## Background

Thyroid carcinoma (TC) is the most common type of endocrine malignancy, for which incidence has grown rapidly worldwide during the past few decades [1, 2]. Owing to biological characteristics and effective therapeutic responses, TC has a favorable long-term prognosis, with an average 10-year overall survival of approximately 90% [3, 4]. However, the overall prognosis worsens significantly once patients develop distant metastasis (DM), as the 10-year survival drops to 40% [5–7]. Even more noteworthy, approximately 70% of TC patients with DM die within four years of diagnosis [8].

Bone metastasis (BM) is a typical metastatic pattern for TC patients. It was reported that BM occur in about 4% of all TC patients [9], and the 5- and 10-year survival rates of TC patients with BM are 61 and 27%, respectively [10]. The majority of thyroid carcinoma metastases are asymptomatic and are detected only upon systemic surveillance or a full-body metastatic examination of malignant thyroid nodules. It is extremely rare to present with symptomatic DM as the sole initial manifestation in the absence of neck swelling [11]. Due to the low incidence of BM and its asymptomatic nature, the work-up regarding BM is mostly ignored during the primary diagnoses of TC patients. Patients are often advised to perform whole-body nuclear medicine bone scanning or PET-CT only when they develop suspicious symptoms of skeletal-related events (SREs). Azeez Farooki et al. reported that the median SRE time was 5 months after BM [6]. At this time, most of patients with TC have likely developed the advanced stage or multiple metastases have occurred, which means the optimal chance of treatment for TC patients will be missed. In 2019, Kim H et al. conducted a retrospective study to evaluate the usefulness of early detection in asymptomatic DM in patients with TC and found that early identification had a significant positive impact on survival outcomes for asymptomatic DM patients with TC after 2004 [12]. A clinical risk model of predicting BM appears to be a helpful tool to clarify how likely a TC patient is to suffer from BM and identify those TC patients with high risk of BM and who should be advised to perform more individualized and purposeful inspection and surveillance plans. Published studies have identified many risk factors of BM including age, histological type, tumour size, marked hypoechogenicity and nodule-in-nodule appearance [8, 13–15]. Nevertheless, no research has focused on the development of an ideal model for predicting BM in TC, which means that the probability of BM cannot be quantified.

Therefore, based on the population-based data from the Surveillance, Epidemiology, and End Results (SEER) database, we aimed to develop and validate a nomogram for predicting the risk of BM in newly diagnosed TC patients. This study might help to further provide more individualized clinical decision guidelines and the rational allocation of health resources.

## Method

### Study population

**Fig. 1:**
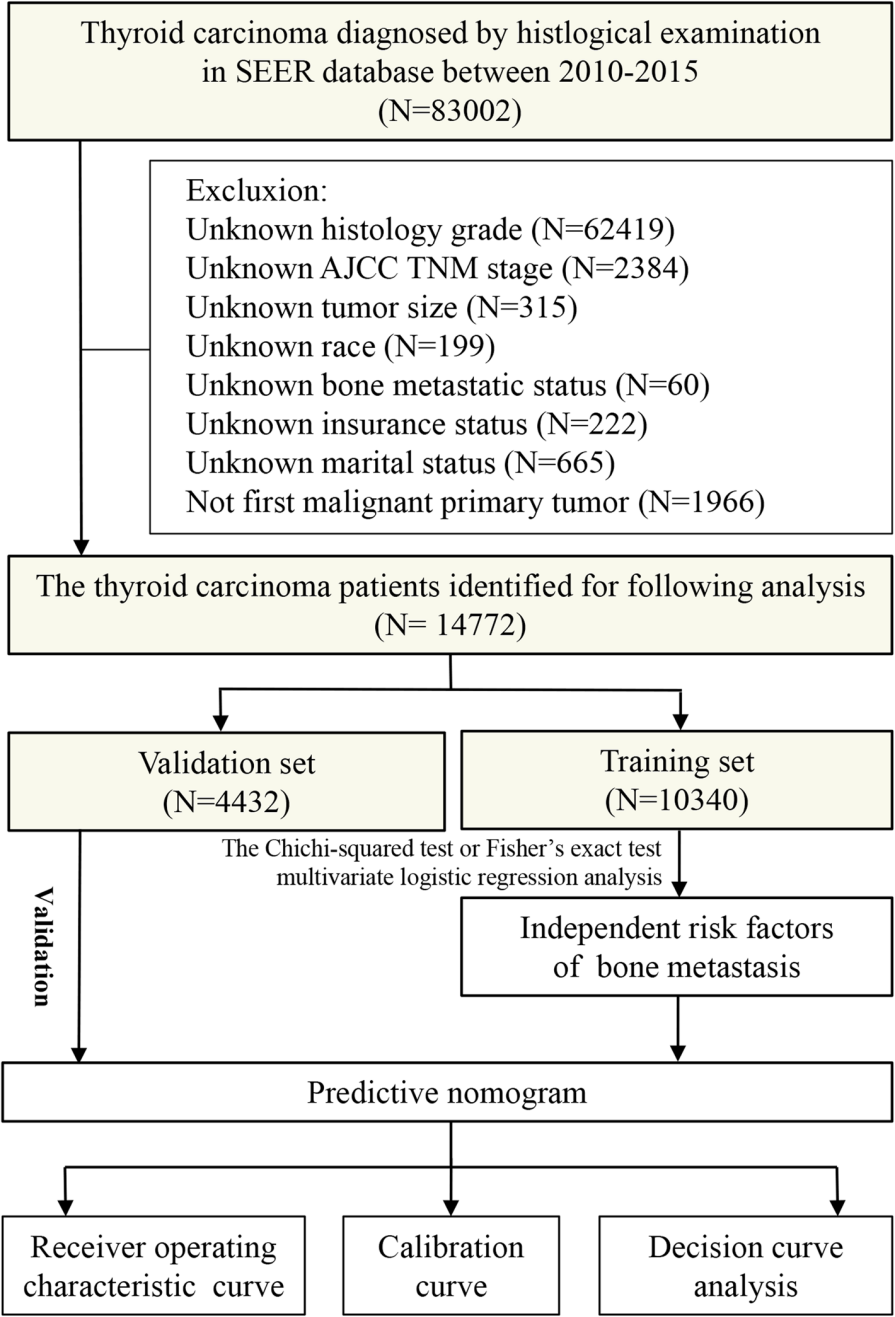
Flow diagram of the analytic cohort selected from the SEER database. Based on the inclusion and exclusion criteria, 14,772 patients were included in this study. After random assignment, 10,340 and 4432 patients were assigned into the training set and the validation set, respectively

The subjects included in the present study were obtained from the Surveillance, Epidemiology, and End Results (SEER) Program. Patient data were downloaded from the SEER∗Stat 8.3.6 Database. We limited this study to between 2010 and 2015 because the information about site-specific metastasis was only available from 2010 and onward. The TC patients included in our research were diagnosed by histological examination, and BM was diagnosed by imaging examination and/or pathological examination. Meanwhile, the exclusion criteria were as follows: (1) the information of race, tumor size, grade, T stage, N stage, bone metastatic status, insurance status and marital status was unknown; (2) TC was not the first tumor. Because the SEER database does not release *personal* identification *information, ethical approval and consent were not required for this study.* Figure 1*displays the flow chart of the patient selection procedure in this study. All included cases were staged using the 7th edition of the AJCC TNM staging system.*

### Data selection

In the present study, 11 variables were included to identify the independent risk factors of BM from TC. The demographic variables included sex, race, age at diagnosis, insurance status and marital status. The clinicopathological variables included histology, grade, T stage, N stage and tumor size, laterality, Histology was classified into four categories with the following IDO-O-3 codes: “8340. 8341.8342.8344.8260. Papillary thyroid carcinoma (PTC)”; “8330. 8331. 8335. Follicular thyroid carcinoma (FTC)”; “8020.8021.8030. 8032. Anaplastic thyroid carcinoma (ATC)”; and “8510.Medullary thyroid carcinoma (MTC)”. “Unmarried”, “separated”, “single”, “widow” and “divorced” were included in the unmarried group and the“ any medicaid”, “insured” and “insured/no specific” were included in the insurance group. All methods were performed in accordance with the relevant guidelines of the SEER database.

### Statistical analyses

All statistical analysis in our research was performed in R software (version 3.6.1, R Foundation for Statistical Computing, Austria) (http://www.r-project.org/) and SPSS (version 25, IBM, USA) [16, 17]. The process of classifying patients in the training and validation sets was completely randomized and was performed in R software. The chi-squared test was used to compare variables between the training set and the testing set. In the univariate analysis, the chi-squared test or Fisher’s exact test was used to compared the variables between patients with BM and without BM. Variables with *p* value< 0.05 in the univariate analysis were further integrated into the multivariate logistic regression analysis. Then, the independent risk factors of BM in TC patients were identified. Based on that, a nomogram was established by rms package in R. Meanwhile, the performance of prediction and discrimination was assessed by Harrell’s concordance index (C-index) which was calculated using the function concordance index. The value of the C-index should range from 0.5 to 1.0, with 0.5 indicating random chance and 1.0 indicating a perfectly corrected discrimination [18]. The receiver operating characteristic (ROC) curve was plotted to show the prediction power of each risk factor and the independent BM-related factors combined model, and the area under curve (AUC) value was also listed. Higher AUC presented a higher prediction power. Calibration curves and decision curve analysis (DCA) were furthermore used to evaluate the clinical value of the nomogram [19]. *P* value< 0.05 (two-sided) was considered as statistically significant.

## Results

### Clinicopathologic characteristics

According to the inclusion and exclusion criteria, 14,772 patients were finally included to identify the risk factors of BM in TC patients. Among these, 120 cases (0.8%) had BM at initial diagnosis and 14,652 cases (99.2%) were without BM. All patients were classified into the training set (10,340 cases) and the validation set (4432 cases), with a ratio of approximately 7:3. In the training set, 24.2% patients were male, and the majority was White in race distribution (80.31%). For grade, 79.9% TC patients were Grade I. The most common T stage and N stage were T1(55.4%) and N0(74.3%), respectively. The demographic and clinicopathological information for all patients is shown in Table 1.

**Table 1:** Clinical and pathological features of 14,772 TC patients

Variables	Training cohort(*N* = 10,340)		Validation cohort(*N* = 4432)		*P* value
	Without BM(*N* = 10,268)	With BM(*N* = 72)	Without BM(*N* = 4384)	With BM(*N* = 48)	
**Age (years)**					**0.757**
<50	5430 (52.9%)	10 (13.9%)	2333 (53.2%)	11 (2.3%)	
≥ 50	4838 (47.1%)	62 (86.1%)	2051 (46.8%)	37 (97.7%)	
**Race**					**0.790**
Black	751 (7.3%)	16 (22.2%)	334 (7.6%)	7 (14.6%)	
Other ^a^	1262 (12.3%)	7 (9.7%)	528 (12.0%)	5 (10.4%)	
White	8255 (80.4%)	49 (68.1%)	3522 (80.4%)	36 (75.0%)	
**Sex**					**0.743**
Female	7802 (76.0%)	39 (54.2%)	3348 (76.4%)	24 (50.0%)	
Male	2466 (24.0%)	33 (45.8%)	1036 (23.6%)	24 (50.0%)	
**Histology**					**0.368**
ATC	203 (2.0%)	24 (33.3%)	68 (1.6%)	11 (22.9%)	
FTC	642 (6.3%)	16 (22.2%)	272 (6.2%)	13 (27.1%)	
MTC	82 (0.8%)	2 (2.8%)	30 (0.7%)	1 (2.1%)	
PTC	9341 (91.0%)	30 (41.7%)	4014 (91.5%)	23 (47.9%)	
**Grade**					**0.092**
Grade I	8247 (80.3%)	19 (26.3%)	3525 (80.4%)	21 (43.8%)	
Grade II	1462 (14.2%)	12 (16.7%)	636 (14.5%)	5 (10.4%)	
Grade III	272 (2.6%)	10 (13.9%)	129 (2.9%)	9 (18.8%)	
Grade IV	287 (2.8%)	31 (43.1%)	94 (2.2%)	13 (27.0%)	
**Laterality**					**0.806**
Unilateral	10,201 (99.3%)	70 (97.2%)	4356 (99.4%)	48 (100.0%)	
Bilateral	67 (0.7%)	2 (2.8%)	28 (0.6%)	0 (0.0%)	
**T stage**					**0.317**
T1	5740 (55.9%)	4 (5.5%)	2460 (56.1%)	10 (20.8%)	
T2	1789 (17.4%)	8 (11.1%)	795 (18.1%)	6 (12.5%)	
T3	2165 (21.1%)	21 (29.2%)	916 (20.9%)	12 (25.0%)	
T4	574 (5.6%)	39 (54.2%)	213 (4.9%)	20 (41.7%)	
**N Stage**					**0.252**
N0	7650 (74.5%)	35 (48.6%)	3231 (73.7%)	23 (47.9%)	
N1	2618 (25.5%)	37 (51.4%)	1153 (26.3%)	25 (52.1%)	
**Tumor size**					**0.569**
<2 cm	6216 (60.5%)	8 (11.1%)	2643 (60.3%)	12 (25.0%)	
2–4 cm	1244 (12.1%)	45 (62.5%)	505 (11.5%)	28 (58.3%)	
>4 cm	2808 (27.3%)	19 (26.4%)	1236 (28.2%)	8 (16.7%)	
**Insurance status**					**0.924**
Insured	10,019 (97.6%)	71 (98.6%)	4280 (97.6%)	46 (95.8%)	
Uninsured	249 (2.4%)	1 (1.4%)	104 (2.4%)	2 (4.2%)	
**Marital status**					**0.927**
Married	6606 (64.3%)	42 (58.3%)	2821 (64.3%)	32 (66.7%)	
Unmarried	3662 (35.7%)	30 (41.7%)	1563 (35.7%)	16 (33.3%)	

Data notes: ^a^ Includes: American Indian, native Alaskan and Asian, Pacific Islander; *PTC* papillary thyroid carcinoma, *FTC* Follicular thyroid carcinoma, *ATC* Anaplastic thyroid carcinoma, *MTC* Medullary thyroid carcinoma

### Independent risk factors for BM from TC

To investigate the independent BM-related factors, univariate analysis was performed. The results of univariate analysis are illustrated in Table 2. Several variables showed significant differences between patients with and without BM, including age, race, sex, tumor size, histology, grade, T stage and N stage, (All *p* value< 0.05). Then, the multivariate logistic regression analysis (forward LR) was performed, and six variables were determined to be independent risk factors, including age at diagnosis(*P* < 0.001), race(*P* < 0.001), grade(*P* = 0.001), and, histology(*P* = 0.013), T stage(*P* < 0.001) and *N* stage(*P* = 0.048). (Table 3).

**Table 2:** Univariate analysis of BM-related variables for TC patients in training set

Variables	Without BM(*N* = 10,268)	With BM(*N* = 72)	*P* value
**Age (years)**			<0.001
<50	5430 (52.9%)	10 (13.9%)	
≥ 50	4838 (47.1%)	62 (86.1%)	
**Race**			<0.001
Black	751 (7.3%)	16 (22.2%)	
Other ^a^	1262 (12.3%)	7 (9.7%)	
White	8255 (80.4%)	49 (68.1%)	
**Sex**			<0.001
Female	7802 (76.0%)	39 (54.2%)	
Male	2466 (24.0%)	33 (45.8%)	
**Histology**			<0.001
ATC	203 (2.0%)	24 (33.3%)	
FTC	642 (6.3%)	16 (22.2%)	
MTC	82 (0.8%)	2 (2.8%)	
PTC	9341 (91.0%)	30 (41.7%)	
**Grade**			<0.001
Grade I	8247 (80.3%)	19 (26.3%)	
Grade II	1462 (14.2%)	12 (16.7%)	
Grade III	272 (2.6%)	10 (13.9%)	
Grade IV	287 (2.8%)	31 (43.1%)	
**Laterality**			0.083
Unilateral	10,201 (99.3%)	70 (97.2%)	
Bilateral	67 (0.7%)	2 (2.8%)	
**T stage**			<0.001
T1	5740 (55.9%)	4 (5.5%)	
T2	1789 (17.4%)	8 (11.1%)	
T3	2165 (21.1%)	21 (29.2%)	
T4	574 (5.6%)	39 (54.2%)	
**N stage**			<0.001
N0	7650 (74.5%)	35 (48.6%)	
N1	2618 (25.5%)	37 (51.4%)	
**Tumor size**			<0.001
<2 cm	6216 (60.5%)	8 (11.1%)	
2–4 cm	1244 (12.1%)	45 (62.5%)	
>4 cm	2808 (27.3%)	19 (26.4%)	
**Insurance status**			0.853
Insured	10,019 (97.6%)	71 (98.6%)	
Uninsured	249 (2.4%)	1 (1.4%)	
**Marital status**			0.289
Married	6606 (64.3%)	42 (58.3%)	
Unmarried	3662 (35.7%)	30 (41.7%)	

Data notes: ^a^ Includes: American Indian, native Alaskan and Asian, Pacific Islander; *PTC* papillary thyroid carcinoma, *FTC* Follicular thyroid carcinoma, *ATC* Anaplastic thyroid carcinoma, *MTC* Medullary thyroid carcinoma

**Table 3:** Multivariate logistic regression analysis of independent predictors for BM in TC patients

Variables	Multivariate logistic regression analysis	
	OR (95% CI)	*P* value
**Age (years)**		
<50	Reference	
≥ 50	3.350 (1.641–6.840)	<0.001
**Race**		
Black	Reference	
Other ^a^	0.328 (0.128–0.837)	0.020
White	0.331 (0.179–0.612)	<0.001
**Histology**		
ATC	Reference	
FTC	3.976 (1.331–11.880)	0.013
MTC	0.823 (0.149–4.549)	0.823
PTC	0.728 (0.292–1.816)	0.728
**Grade**		
Grade I	Reference	
Grade II	2.320 (1.096–4.908)	0.028
Grade III	4.701 (1.959–11.286)	0.001
Grade IV	7.068 (2.111–23.672)	0.002
**T stage**		
T1	Reference	
T2	3.689 (1.065–12.780)	0.040
T3	6.819 (2.233–20.823)	0.001
T4	10.978 (2.898–41.584)	<0.001
**N stage**		
N0	Reference	
N1	1.768 (1.005–3.109)	0.048

Data notes: ^a^ Includes: American Indian, native Alaskan and Asian, Pacific Islander; *PTC* papillary thyroid carcinoma, *FTC* Follicular thyroid carcinoma, *ATC* Anaplastic thyroid carcinoma, *MTC* Medullary thyroid carcinoma

### Development and validation of the nomogram

**Fig. 2:**
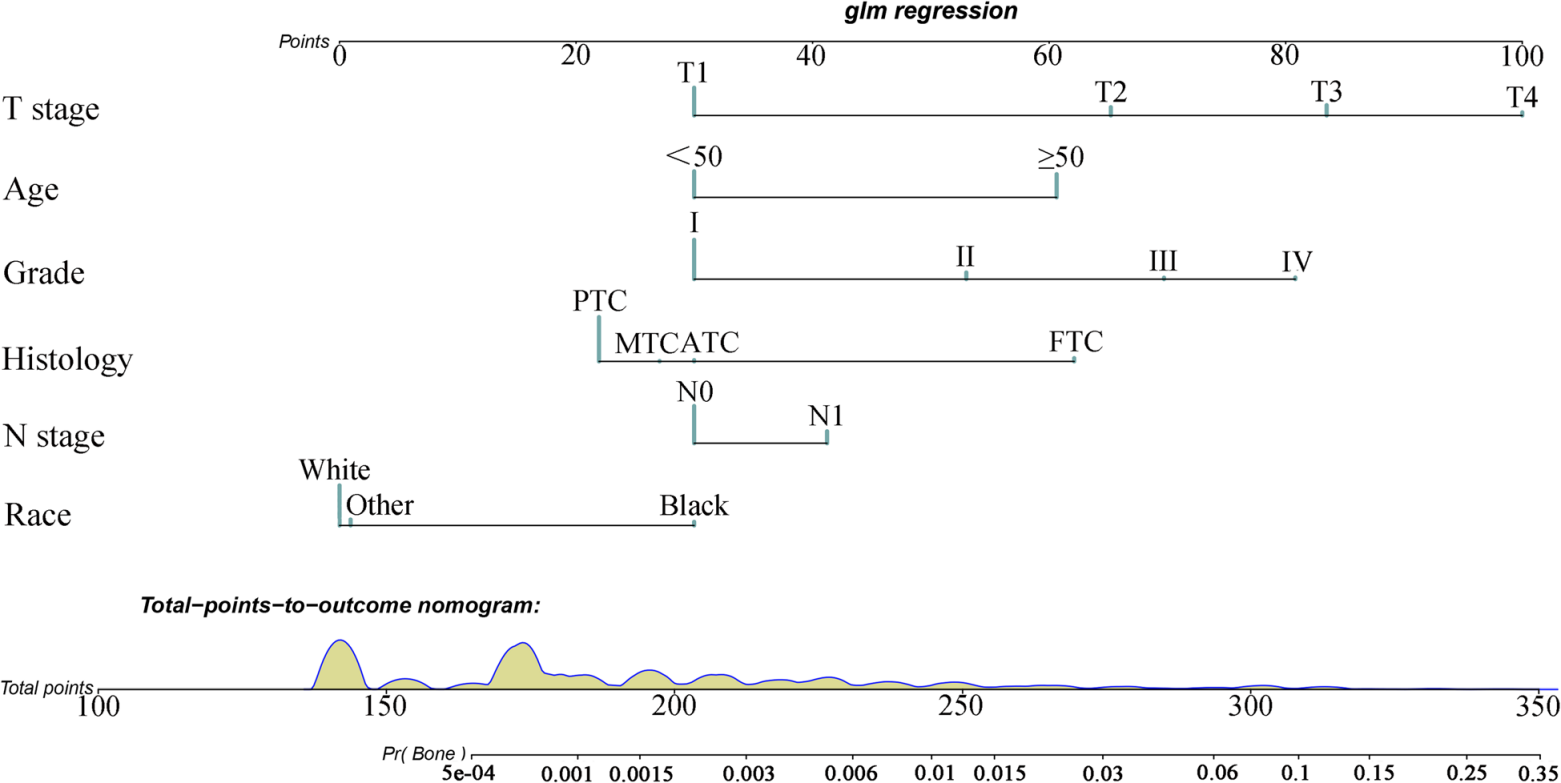
A nomogram for predicting risk of bone metastasis from thyroid carcinoma. The values of each independent risk factor for individual patients are located along the variable axes, and a line is drawn upward to the point axis to determine the number of points assigned for each variable. There was a total points line at the bottom of the nomogram, and each variable score was summed to give the total points. Then, a vertical line was drawn from the total points scale to the BM axis to obtain the probability

**Fig. 3:**
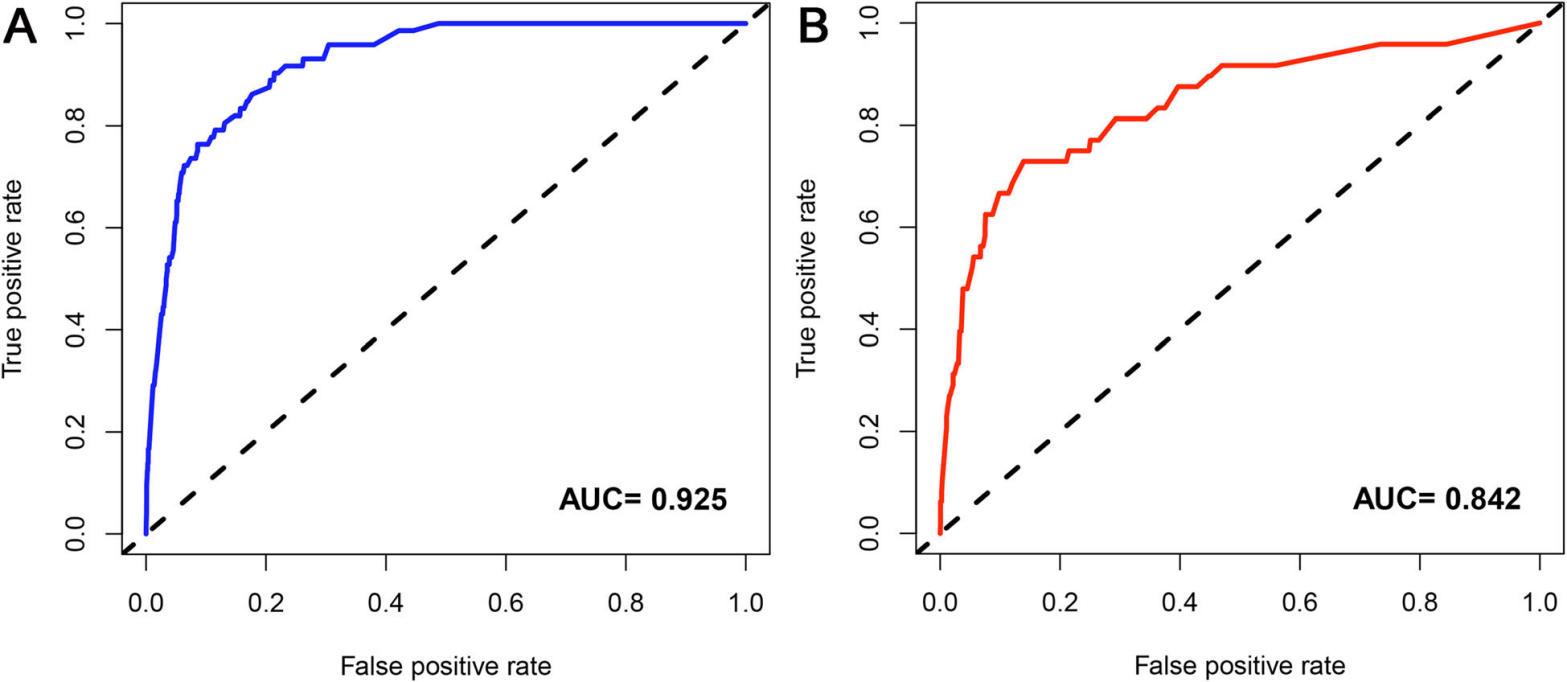
Receiver operating characteristic (ROC) curves and area under the curve (AUC) of the nomogram for predicting bone metastasis from thyroid carcinoma in the training set **a** and validation set **b**

**Fig. 4:**
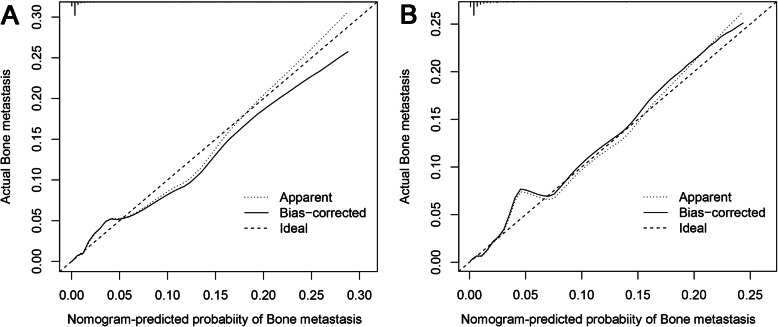
Comparison of the area under the receiver operating characteristic curve between the nomogram and each independent predictor in the training set **a** and validation set **b**. The AUC of the combined model was the highest, indicating that the combined model showed the highest predictive power of BM at diagnosis of TC

**Fig. 5:**
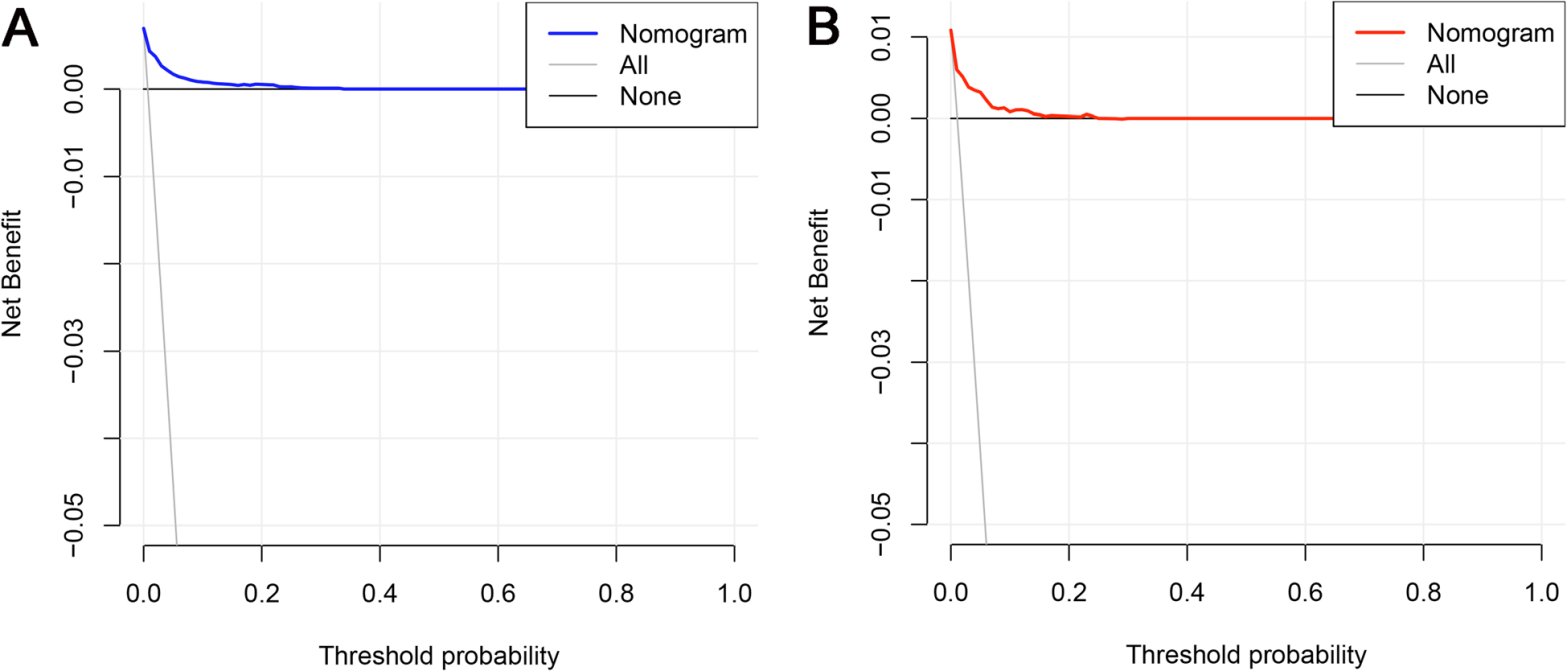
Calibration curves of the nomogram for predicting bone metastasis from thyroid carcinoma in the training set **a** and validation set **b**. The X-axis represents the nomogram-predicted probability of BM, and the Y-axis represents the actual probability of BM. Plots along the 45-degree line indicate a perfect calibration model in which the predicted probabilities are identical to the actual outcomes

**Fig. 6:**
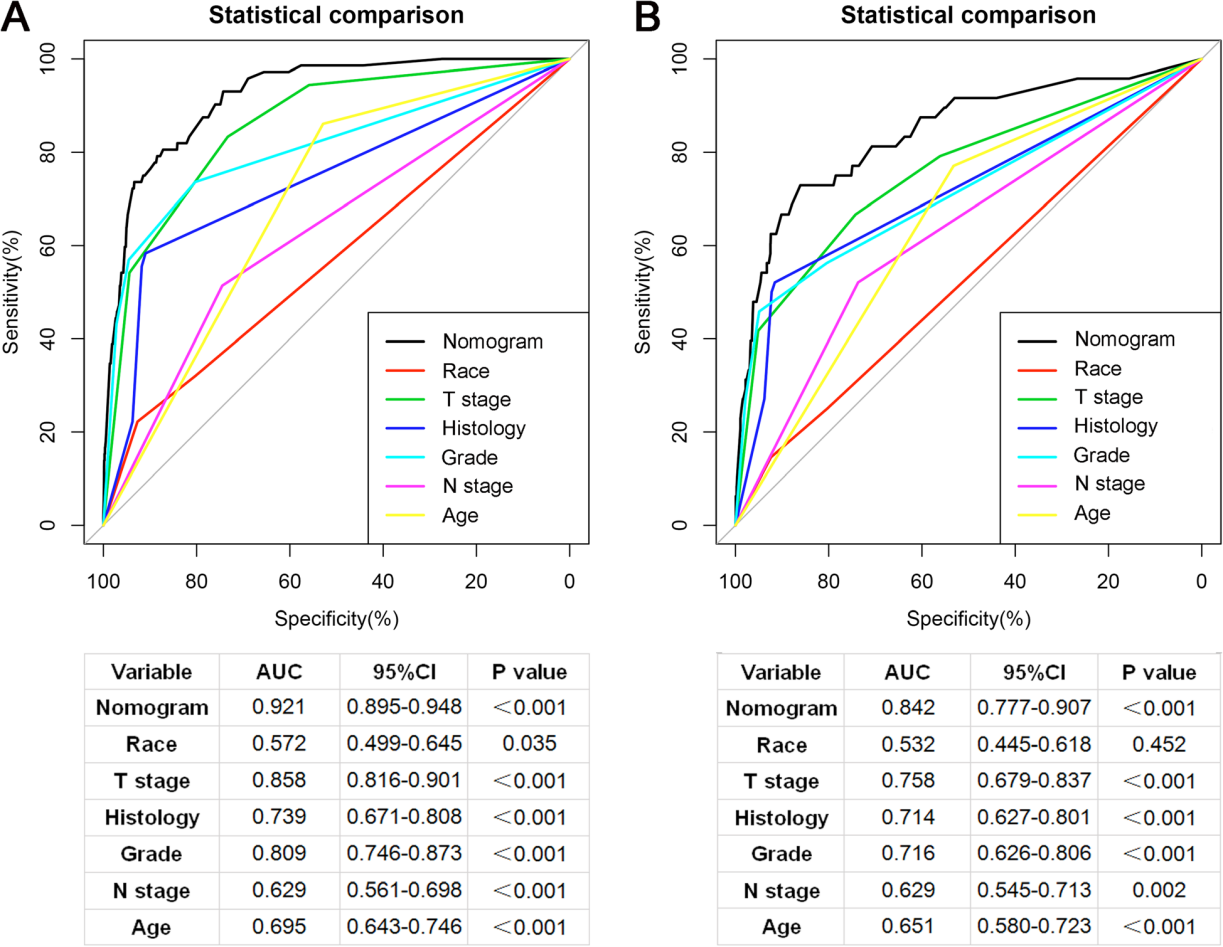
Decision curve analysis (DCA) of the nomogram for predicting bone metastasis from thyroid carcinoma in the training set **a** and validation set **b**. This nomogram shows a notable positive net benefit, indicating that it has a good clinical utility in predicting the risk of BM in patients with TC

Based on the independent risk factors, a nomogram was established (Fig. 2). The total score value of each individual patient was obtained by adding the corresponding scores of different categories of each independent risk factor, and then corresponding total points scale represented the probability of BM of this patient. The C-indexes in the training set and validation set were 0.925(95%CI,0.895–0.948) and 0.842(95%CI,0.777–0.907), respectively, which indicates that the nomogram performs well in predicting BM of TC patients. Meanwhile, ROC curves of both the training set and validation set were generated and illustrated in Fig. 3. Remarkably, the ROC curves of each independent predictor were also generated (Fig. 4). The AUC of the combined model was the highest, which indicated the combined model showed the highest prediction power of BM at diagnosis. In addition, the favorable calibration curve of our nomogram is shown in Fig. 5, which indicated that the prediction by the nomogram is highly consistent with the actual observations. Finally, DCA indicated that this nomogram can serve as an excellent diagnostic tool for predicting BM in newly diagnosed TC patients (Fig. 6).

## Discussion

BM frequently deteriorates the quality of life by inducing severe bone pain, spinal cord compression, pathological fracture and other SREs. Farooki A et al. have found that approximately 78% TC patients with BM develop at least one SRE [6]. Orita Y et al. observed 52 DTC patients with BM that constituted 3.7% of 1398 patients [20]. A recent study also reported a similar result wherein BM occurred in 3.9% (1173 cases) of the TC patients [9]. Here, we found that the proportion of BM incidence (0.8%) in TC patients was much less than previous reports. This can be attributed to the rare incidence of synchronous BM recorded in the SEER database instead of metachronous BM in other studies. Synchronous BM diagnosed in TC patients is very rare, in other words, a majority of BM develops during clinical follow-up after the primary diagnosis of TC. Iñiguez-Ariza NM and colleagues conducted a systematic review and suggested that the natural disease course of BM from TC may vary by demographic factor and tumor characteristics [8]. The early identification of BM at the initial diagnosis of TC has great significance for receiving appropriate treatment and improving prognosis. To better address this issue, we used a population-based database to identify independent risk factors for BM and developed a prediction model based on demographics and tumor characteristics to predict the risk of BM in patients with newly diagnosed TC.

In the present study, we found six independent predictors associated with BM, including age at diagnosis, histology, grade, T stage, N stage and race. More importantly, we developed and validated a diagnostic nomogram for the purpose of predicting BM in newly diagnosed TC patients. The total score can be calculated through collecting the information of several variables on the nomogram of every TC patient. Then, the risk of BM can be identified from the nomogram with ease. Meanwhile, this predictive model demonstrated excellent performance in the risk assessment of BM from TC, which will make personalized medical decision-making and surveillance more accurate. Although SRE has long been identified as a sign of bone metastatic disease, it is unreasonable to consider BM and perform targeted investigations in thyroid cancer patients only when they have symptoms of bone involvement. Thus, proactive attention should be given to patients with TC who have been identified by nomogram as having a high risk of BM. In a previous study, Goffredo P et al. found that the risk of DM for younger TC patients was significantly lower compared with elderly patients [21]. Similarly, age at diagnosis was identified as an independent predictor of BM in TC patients in our study(*P* < 0.001). More evidence now suggests that the biological characteristics of tumors play a crucial role in disease progression, which could be closely associated with the initiation and development of BM. Vuong HG and colleagues performed a meta-analysis and found that tumor size, multifocality, vascular invasion (VI), extrathyroidal extension (ETE), lymph node metastasis (LNM), and lateral LNM were associated with significant risks for DM [15]. Liu Z et al. suggested that LNM has a synergic effect with either follicular thyroid histology or larger tumor size for a higher risk of DM, which is important for predicting and diagnosing DM [22]. Here, we found that T stage and N stage were independent risk factors associated with BM in TC patients. We also found that patients with poorly or undifferentiated tumors were more prone to have BM. This might be because cancer cells have the capability of invading surrounding tissues, capillaries and lymphatics with stronger growth potential to develop early metastasis. These findings are in agreement with the research of other scholars [23]. TC was highly heterogeneous in terms of its molecular and clinical properties, and consist of four main subtypes that are associated with different tendencies of BM. As previously noted by Do M Y et al. [24], although PTC is the most frequent type of TC, FTC is more prone to BM. The results of multivariate logistic regression analysis showed that the risk of BM was highest in FTC. It is therefore possible and reasonable that blood vessel invasion is more common in FTC than in PTC.

There are several strengths of this study. First of all, it was a population-based study with a large sample size that included all types of TC, and the results of this study are therefore of good representative and clinical guidance value. Besides, at the molecular level, osteocalcin, cDNA and the expression of focal adhesion kinase (FAK), Integrin αvβ3 and cDNA were thought to be associated with BM from TC [25–28]. However, these biomarkers were inconvenient and impractical to apply promptly to clinical decision-making. The independent risk factors identified in this study are common clinical predictors, which can be easily accessed in routine clinical practice routine clinical practice. Most importantly, a nomogram with excellent performance was developed to identify the individual risk of BM from TC by combining all independent predictors, which means that the probability of BM can be quantified.

Despite these advantages, this study still faces several limitations. First, as a retrospective study, potential selection bias was inevitable. Second, the information recorded in the SEER database was descriptive of the disease at the initial diagnosis, which indicates that treatment data cannot be included in the prediction analysis of BM from TC. Third, the nomogram provided a relative reference for clinical doctors. Some other factors that are relevant to the risk of BM in TC are likely to exist in the clinical environment.

## Conclusion

Our study suggests that age, race, histology, grade, T stage and N stage are independent BM-related risk factors for TC. Furthermore, the predictive nomogram we created is expected to be a convenient, personalized and visual clinical tool for risk assessment of BM in newly diagnosed TC.

## Data Availability

The datasets generated and/or analyzed during the current study are available in the SEER database (*https://seer.cancer.gov/*).

## Abbreviations

TC: Thyroid carcinoma
TCBM: Thyroid carcinoma with bone metastasis
BM: Bone metastasis
SEER: Surveillance, Epidemiology and End Results
ROC: Receiver operating characteristic curve
AUC: Area under the curve
DCA: Decision curve analyses
SRE: Skeleton-related event
PTC: Papillary thyroid carcinoma
FTC: Follicular thyroid carcinoma
ATC: Anaplastic thyroid carcinoma
MTC: Medullary thyroid carcinoma
VI: Vascular invasion
ETE: Extrathyroidal extension
LNM: Lymph node metastasis
